# 600. Through the Eyes of the Frontline: Providers’ Perceptions on Pediatric Readiness

**DOI:** 10.1093/jbcr/irag033.404

**Published:** 2026-04-06

**Authors:** Carey K Lamphier, Laura S Johnson, Rahima Ladak, Pamela Vanderberg, Alexandra K Monroe

**Affiliations:** Grady Memorial Hospital, Atlanta, GA; Grady Memorial Hospital / Emory School of Medicine / Morehouse School of Medicine, Atlanta, GA; Grady Memorial Hospital, Atlanta, GA; Grady Memorial Hospital, Atlanta, GA; Emory University/Grady Memorial Hospital/Children's Healthcare of Atlanta, Atlanta, GA

## Abstract

**Introduction:**

The care of acutely ill and injured children presents significant emotional and psychological challenges for healthcare providers, especially when they present to primary adult settings. The low frequency high stakes nature of such events can leave frontline staff feeling underprepared and overwhelmed. Understanding frontline providers perspectives on pediatric preparedness, training, and hospital system support is critical to identifying gaps and improving the pediatric readiness at our large volume, Verified Burn and Trauma Center.

**Methods:**

Physician and nursing leadership from the burn center and trauma center collaborated to evaluate frontline provider perspectives on pediatric readiness through a mixed method, community based participatory research approach. Institutional Review Board (IRB) approval was obtained to collect survey responses from various provider groups, including patient care technicians, nurses, physicians, pharmacists, and respiratory therapists. A REDCap survey consisting of 17 quantitative and qualitative questions was distributed to frontline staff to identify both strengths and gaps in pediatric care within our institution. Survey participants were recruited from Broselow Cart education sessions, Pediatric Mock Codes, and staff meetings. Recruitment efforts targeted units actively involved in the care of pediatric patients, such as the Burn Unit and Trauma Center.

**Results:**

Of 45 unique respondents, 48% were from the Burn Unit or Trauma Center. Since pediatric burn patients are typically first seen in the Trauma Center, this subgroup was analyzed separately. Within it, 82% were female, 78% were nurses, 53% had less than 10 years of medical experience, and 13% had more than 25 years.

50% of respondents identified "Staff Education" as the top facilitator of safe pediatric care, while 37% also saw it as a barrier. Additionally, 32% cited “Expertise” as a barrier, consistent across both centers. Trauma Center staff highlighted equipment as being the biggest gap; the Burn Unit did not. The overall mean comfort level with pediatric care was 4.05 ± 1.52 (scale of 1–7).

**Conclusions:**

Using Community Based Participatory Research, we identified both the perceived facilitators and gaps in delivering high-quality pediatric care. Guided by theories of change like Maslow’s Hierarchy of Needs, we found that the most immediate improvements can be made in equipment, policies, procedures, and education. These are addressable through collaboration with supply teams, policy updates, and expanded education programs. In contrast, developing expertise, at the top of the hierarchy, requires ongoing investment in staff education and will improve over time.

**Applicability of Research to Practice:**

Build and sustain pediatric education that promotes both patient physical safety and staff psychological safety.

**Funding for the study:**

Seed grant funding.

